# Cognitive function in very old men does not correlate to biomarkers of Alzheimer’s disease

**DOI:** 10.1186/s12877-017-0601-6

**Published:** 2017-09-08

**Authors:** V. Velickaite, V. Giedraitis, K. Ström, I. Alafuzoff, H. Zetterberg, L. Lannfelt, L. Kilander, E-M. Larsson, M. Ingelsson

**Affiliations:** 1 Department of Surgical Sciences, Radiology, Uppsala University, Uppsala, Sweden; 2 Department of Public Health and Caring Sciences/Geriatrics, Uppsala University, Uppsala, Sweden; 3 Department of Immunology, Genetics and Pathology, Uppsala University, Uppsala, Sweden; 4 Department of Pathology Uppsala University Hospital, Uppsala, Sweden; 5 Department of Psychiatry and Neurochemistry, the Sahlgrenska Academy at the University of Gothenburg, Mölndal, Sweden; 6 Clinical Neurochemistry Laboratory, Sahlgrenska University Hospital, Mölndal, Sweden; 7 Department of Molecular Neuroscience, UCL Institute of Neurology, Queen Square, London, UK

**Keywords:** Brain atrophy, Cognitive performance, CSF biomarkers, Neuropathology, AD biomarkers, Advanced age

## Abstract

**Background:**

The Alzheimer’s disease (AD) brain displays atrophy with amyloid-β (Aβ) and tau deposition, whereas decreased Aβ42 and increased tau are measured in cerebrospinal fluid (CSF). The aim of this study was to relate cognitive performance to the degree of brain atrophy, CSF biomarker levels and neuropathology in a cohort of aged men.

**Methods:**

Fifty-eight 86–92-year-old men from the Uppsala Longitudinal Study of Adult Men (ULSAM) cohort underwent cognitive testing, brain computed tomography and lumbar puncture. Atrophy was graded with established scales. Concentrations of CSF Aβ42, t-tau and p-tau were measured by ELISA. Thirteen brains were examined *post mortem*.

**Results:**

Forty-six of the individuals were considered non-demented, whereas twelve were diagnosed with dementia, either at baseline (*n* = 4) or during follow-up (*n* = 8). When comparing subjects with and without dementia, there were no differences in the degree of atrophy, although the mini mental state examination (MMSE) scoring correlated weakly with the degree of medial temporal atrophy (MTA) (*p* = 0.04). Moreover, the CSF biomarker levels did not differ significantly between healthy (*n* = 27) and demented (*n* = 8) subjects (median values 715 vs 472 pg/ml for Aβ42, 414 vs 427 pg/ml for t-tau and 63 vs 60 pg/ml for p-tau). Similarly, there were no differences in the biomarker levels between individuals with mild (*n* = 24) and severe (*n* = 11) MTA (median values 643 vs 715 pg/ml for Aβ42, 441 vs 401 pg/ml for t-tau and 64 vs 53 pg/ml for p-tau). Finally, the neuropathological changes did not correlate with any of the other measures.

**Conclusion:**

In this cohort of aged men only a weak correlation could be seen between cognitive performance and MTA, whereas the various neuroradiological, biochemical and neuropathological measures did not correlate with each other. Thus, AD biomarkers seem to be less informative in subjects of an advanced age.

## Background

Cognitive disorders increase exponentially with advancing age. At the age of 80, the prevalence of dementia has been estimated to 10–12% whereas approximately 40% of individuals over 90 years are affected [1]. Although better early-life socioeconomic conditions and treatment of cerebrovascular risk factors may have beneficial effects on brain health, the prevalence of dementia is likely to become even higher in the future due to increasing life expectancy [1].

In Alzheimer’s disease (AD), the most common cause of dementia, amyloid-β (Aβ) plaques and neurofibrillary tangles of tau proteins are found in widespread areas of the *post mortem* brain [2]. However, clinico-pathological studies have demonstrated a lack of correlation between brain function and pathology in aging individuals. Accordingly, some cognitively intact elderly subjects may display abundant protein deposition or cerebrovascular lesions at autopsy whereas a subgroup of patients with cognitive dysfunction may show a relatively mild neuropathology [3–8]. Thus, the relationship between pathological brain lesions and the clinical status seems to be attenuated at advanced ages. In a recent study, when the brain was assessed *post mortem* in almost 300 subjects without neurological impairment, it was demonstrated that approximately half of the subjects (47%) displayed Aβ deposition whereas some degree of tau pathology could be seen in almost all brains (98%) [9]. Thus, the protein deposits may not themselves confer a high degree of toxicity and it has instead been suggested that other pathological alterations, such as soluble prefibrillar species of Aβ and tau, correlate better to the cognitive dysfunction in AD [10].

Similarly, brain imaging of older patients may not be conclusive. The medial temporal lobe atrophy (MTA) or global cortical atrophy (GCA), as visualized by computed tomography (CT) or magnetic resonance imaging (MRI), are changes that indicate AD development [11, 12]. However, such structural changes are commonly seen also in cognitively healthy subjects older than 80 years [13, 14].

The development of cerebrospinal fluid (CSF) biomarkers has been of great importance to identify subjects with mild cognitive impairment that subsequently will convert to AD dementia [15]. However, these markers are not suitable to monitor clinical progression as they remain relatively stable throughout the disease process [16]. Moreover, it has been observed that the predictive and diagnostic value of CSF markers is limited in older populations where a substantial part of apparently cognitively healthy subjects still have an AD CSF profile of decreased Aβ42 together with increased levels of total tau (t-tau) and phospho-tau (p-tau) [17, 18]. In addition, a substantial overlap in the biomarker profile has been demonstrated between AD and non-AD dementia cases [19, 20].

Studies on the relationship between protein pathology, structural abnormalities, CSF biomarkers and cognition in subjects of an advanced age are still scarce. We therefore assessed these aspects on a group of 86–92 year old men from the population-based Uppsala Longitudinal Study of Adult Men (ULSAM) cohort.

## Methods

### Study design and participants

ULSAM represents a longitudinal epidemiological, still ongoing, study based on all available men born 1920–1924 and who were living in Uppsala county in the beginning of the 1970s. These men had all undergone extensive examinations at approximately 50, 60, 70, 77, 82 and 88 years of age [21, 22]. Full screening and official registry data are available in databases, which are being continuously updated (http://www.pubcare.uu.se/ulsam).

Of the 2322 individuals who were initially recruited to the ULSAM study, 354 had been examined at the age of 84–88 (ULSAM-88). Of the ULSAM-88 participants, 198 individuals were considered to be able to visit the hospital. However, 38 subjects were reported to be under Coumadin treatment and were therefore excluded. Thus, a total of 160 remaining ULSAM individuals were invited to the study.

Fifty-eight individuals agreed to participate in the study. Of these, twelve were diagnosed with AD or unspecified dementia, either at baseline (*n* = 4) or during the following four years (*n* = 8). The diagnostic procedures have been previously described in detail [22]. The other 46 subjects were considered to be cognitively healthy and scored >25p in the MMSE. All study subjects except one were genotyped for *APOE* by minisequencing [23].

The participants underwent CT of the brain and cognitive testing by the mini mental state examination (MMSE). In addition, for all subjects enhanced cue recall test (ECRT) data were available from the recently concluded 84–88 years’ examination.

Fifty-two of the 58 subjects accepted to undergo lumbar puncture (LP), although only 35 of these could be completed. Of the twelve demented individuals, CSF samples were obtained from eight whereas 27 samples could be collected from the 46 cognitively healthy subjects. Finally, all 58 subjects were asked for a *post mortem* brain donation and 24 of them gave their informed consent. Of these, 13 brains have so far come to autopsy.

### Cognitive rating

Forty-nine of the participating individuals underwent MMSE [24]. The test was administered to all subjects by the same investigator (KS) prior to the lumbar puncture. Also, as a part of their 88 year follow up examination, 57 of the participants had undergone an enhanced cued recall test from the Seven Minute Screen [25]. Scores from the spontaneous recall were used, which we in the following will refer to as the “object recall test”. This test had been applied 1–4 years before the other assessments.

### CT of the brain

The CT examinations were performed at the Uppsala University Hospital. An 8-slice scanner (General Electric, Boston, MA) or a 64-slice scanner (Siemens Healthengineers, Erlangen, Germany) was used to acquire the images.

**Fig. 1:**
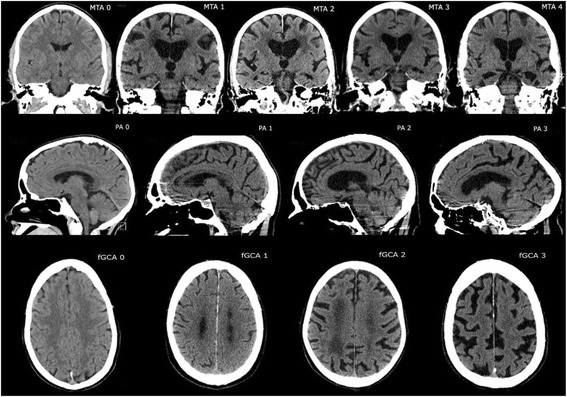
Example images of subjects with different grades of medial temporal lobe (MTA), posterior atrophy (PA) and frontal cortical atrophy (fGCA), according to the different scales applied in this study

The CT images were reformatted to axial, sagittal and coronal planes, with a slice thickness of 4 mm. All images were independently reviewed by two neuroradiologists (VV and EML) who were blinded to cognitive status and CSF findings. The degree of medial temporal lobe atrophy (MTA) was graded using the Scheltens scale [26]. The posterior atrophy (PA) was assessed according to the Koedam scale and the frontal atrophy according to the Pasquier scale for global cortical atrophy (fGCA) [27, 28]. These scales are graded from 0, with no atrophy, to either 3 (Koedam and Pasquier scales) or 4 (Scheltens scale), corresponding to the most severe degree of atrophy (Fig. 1). Also, white matter changes, evaluated according to the Fazekas scale and the presence of lacunar or cortical infarcts, were defined [29, 30]. In cases of disagreement between the neuroradiologists a consensus evaluation was made to reach the final scoring results.

### Lumbar puncture

Lumbar puncture was performed by one investigator (MI) with the patient in a lying position. Twelve milliliters of CSF were collected in polypropylene tubes and samples with a clear visual blood contamination were excluded. The samples were centrifuged and aliquoted in 1.5 ml polypropylene tubes, followed by storage at −70 °C until the analyses.

### CSF measurements

The concentrations of Aβ42, t-tau and p-tau in CSF were measured at the Clinical Neurochemistry Laboratory, University of Gothenburg, Mölndal, Sweden. The measurements were performed by board-certified laboratory technicians, who were blinded to clinical data, using sandwich ELISAs (INNOTEST, Fujirebio, Ghent, Belgium) and procedures accredited by the Swedish Board of Accreditation and Conformity Assessment.

### Neuropathology

Brains from 13 subjects were removed *post mortem*, followed by neuropathological assessment. The left hemisphere was immersed in 4% buffered formalin and after three days cut in 1 cm thick coronal slices. All grossly notable lesions were registered and 16 neuroanatomical regions were sampled [9]. Seven μm thick sections were generated for hematoxylin-eosin (HE) and immunohistochemical (IHC) stains. All 16 neuroanatomical regions were stained applying HE to assess the presence or absence of vascular alterations. The IHC stainings were carried out to visualize altered proteins such as hyperphosphorylated tau (HPτ, clone AT8), Aβ (Aβ, clone 63F), α-synuclein (αS, clone KM51) and phosphorylated transactive DNA binding protein 43 (TDP43, clone 11.9). The assessment of the severity of altered proteins was based on current recommendations: for HPτ - Braak stage ranging from I to VI, for Aβ - Thal phase ranging from 1 to 5 and for αS - Braak stage ranging from 1 to 6 [31–33]. For cerebral amyloid angiopathy (CAA), type 1 or 2 CAA was assigned. Deposition of pTDP43 was assessed in medulla, amygdala and hippocampus and was assigned as positive if any or all of these regions were affected. The level of AD related pathology was assessed as recommended by the National Institute of Aging and Alzheimer’s Association (NIA-AA) and subjects with primary age related tauopathy (PART) were identified [34–36]. All neuropathological slices were reviewed by a neuropathologist (IA).

### Statistics

For the comparison between groups, Mann–Whitney U test or Kruskal–Wallis ANOVA was used. All statistical analyses were performed with STATISTICA v13. Levels of quantitative variables are presented as mean ± SD or median and range. Two-tailed values of *P* < 0.05 were considered statistically significant.

## Results

A general description of the participating subjects, including age, disease status, education, *APOE* status and overall outcome on the cognitive testing is given in Table 1. The median MMSE score was 28 for the whole group (25.5 for those who already had or were subsequently given a dementia diagnosis and 28 for those without a dementia diagnosis). For the object recall test the median score for the whole group was 7 (4.5 for those with and 7 for those without a dementia diagnosis). Only five out of 57 genotyped individuals were *APOE ε*4 carriers (8.8%) and, interestingly, all of them were non-demented. Twelve subjects (21.1%), both demented and cognitively healthy, were carriers of the *APOE ε*2 allele.

**Table 1:** Description of the participants

Age, mean ± SD (range)	88.5 ± 0.9 (86–92)
Demented/non-demented, N	12/46
Computed tomography, N	58
CSF analyses, N	35
*Post mortem* evaluation, N	13
MMSE (*N* = 49), median (range)	28 (19–30)
Non-demented (*N* = 39)	28 (25–30)
Demented (*N* = 10)	25.5 (19–29)
Object recall test (*N* = 57), median (range)	7 (1–12)
Non-demented (*N* = 45)	7 (3–12)
Demented (*N* = 12)	4.5 (1–8)
Education (*N* = 58)	
Primary school (6–7 years), N (%)	29 (50)
Secondary school (8–13 years), N (%)	18 (31)
University (> 13 years), N (%)	11 (19)
*APOE* genotype (*N* = 57)	
ε4 carriers, N (%)	5 (8.8)
ε4 non-carriers, N (%)	52 (91.2)

*SD* standard deviation, *CSF* cerebrospinal fluid, *MMSE* mini mental state examination, *APOE* apolipoprotein E

**Fig. 2:**
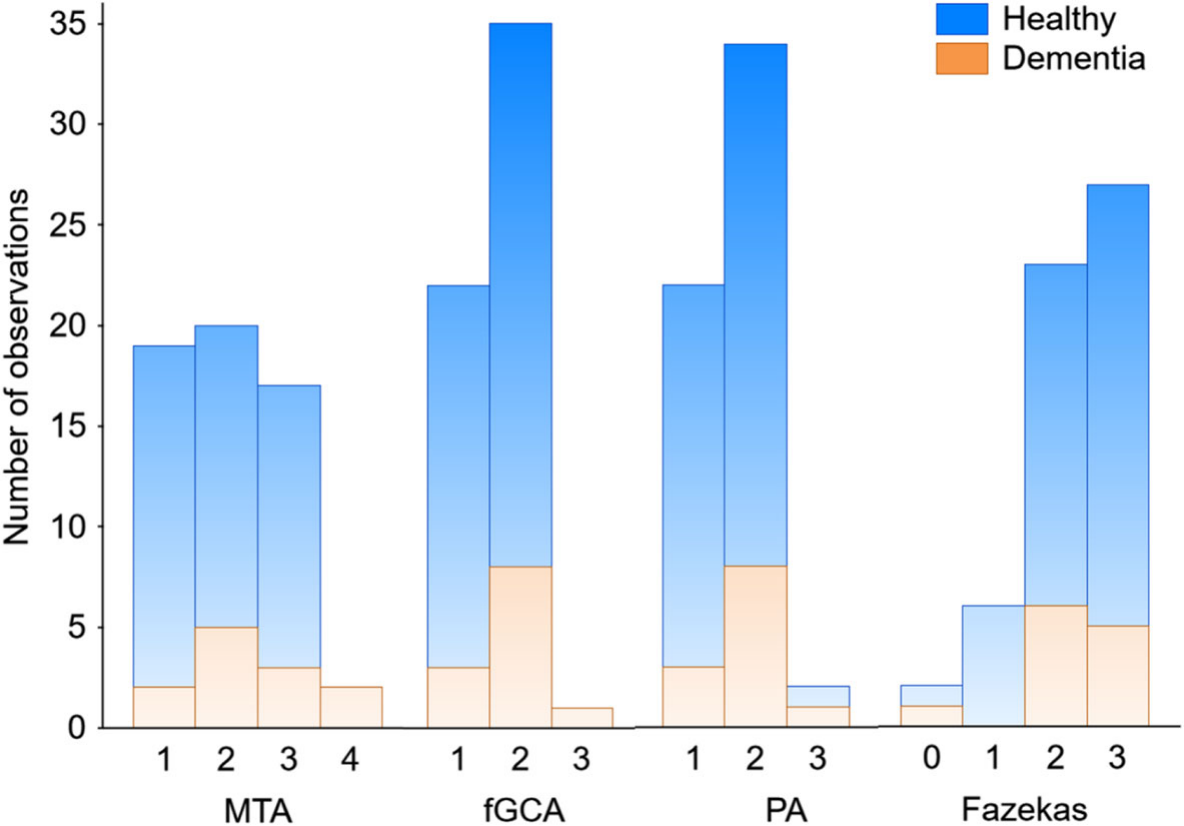
Number of individuals with the different grades of MTA, fGCA, PA and white matter changes (Fazekas)

On CT, the most severe atrophy grades according to the different scales were seen only in very few individuals (Fig. 2). For the majority, there was an equal representation of MTA grades 1, 2 and 3, whereas grade 2 was the most common degree of fGCA and PA (Fig. 2). As for the white matter changes, Fazekas grades 2 and 3 were the most commonly found. When we subdivided the entire group into subjects with no cognitive dysfunction and demented subjects there were no differences in the degree of different atrophy measures (Fig. 2). The extent of medial temporal, global cortical and parietal atrophy was similar regardless of the clinical status. For the degree of white matter changes, there were also no differences between the groups (Fig. 2). The relation between the neuroradiological findings and the clinical status and *APOE* genotypes are shown in Table 2.

**Table 2:** Neuroradiological findings and clinical status, related to *APOE* genotypes

	A*POE*	
	*ε*4 carriers	*ε*4 non-carriers
Total, N (%)	5 (8.8)	52 (91.2)
MTA		
1–2	2 (40)	37 (71)
3–4	3 (60)	15 (29)
fGCA		
1	0 (0)	21 (40)
2–3	5 (100)	31 (60)
PA		
1	0 (0)	21 (40)
2–3	5 (100)	31 (60)
Fazekas		
0_2	3 (60)	27 (52)
3	2 (40)	25 (48)
Infarcts		
Lacunar	3 (60)	24 (48)
None	2 (40)	26 (52)
Clinical status		
Non-demented	5 (11)	40 (89)
Demented	0 (100)	12 (100)

*APOE* apolipoprotein E, *MTA* medial temporal atrophy, *fGCA* frontal global cortical atrophy, *PA* posterior atrophy

**Fig. 3:**
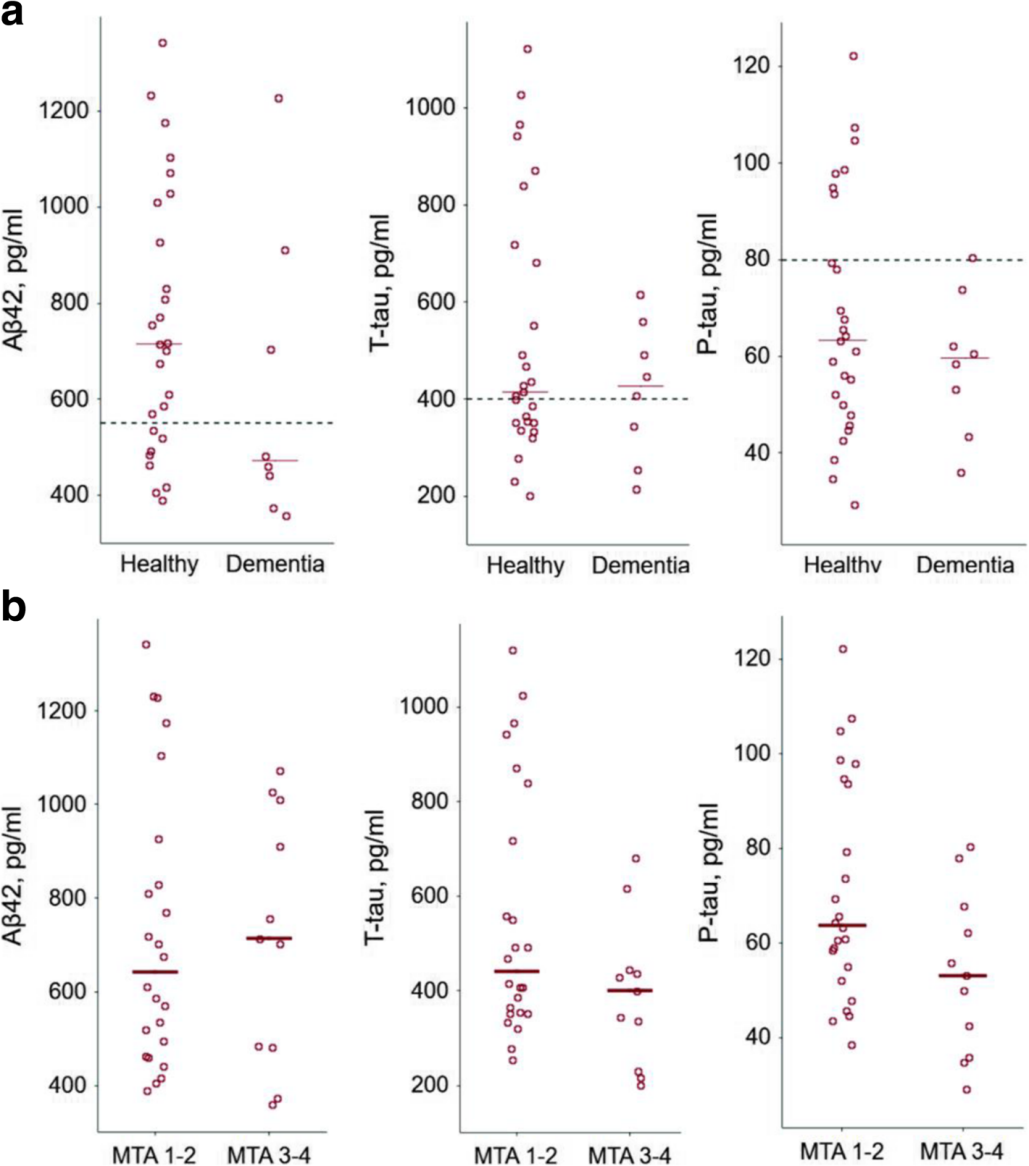
Comparison of t-tau, p-tau and Aβ42 CSF concentrations between different groups. **a** Cognitively healthy (*n* = 27) and demented (*n* = 8) individuals (median levels 715 vs 472 pg/ml for Aβ42, 414 vs 427 pg/ml for t-tau and 63 vs 60 pg/ml for p-tau). **b** Individuals with mild (grades 1–2, *n* = 24) and severe (grades 3–4, *n* = 11) MTA (median levels 643 vs 715 pg/ml for Aβ42, 441 vs 401 pg/ml for t-tau and 64 vs 53 pg/ml for p-tau). No significant differences were observed between the groups for any of these biomarkers. Horizontal line on graphs indicates median levels. Dashed lines represent reference values

The CSF levels of the AD biomarkers ranged from 349 to 1343 pg/ml (median level 703 pg/ml) for Aβ42, 202–1121 pg/ml (median level 414 pg/ml) for t-tau and 29–122 pg/ml (median level 61 pg/ml) for p-tau. When comparing the levels between cognitively intact and demented subjects there were no differences between the groups (Fig. 3a).

Next, we wanted to assess possible associations between the different cognitive, neuroradiological and biochemical markers. For these analyses we combined the individuals with MTA 1 or 2 and those with MTA 3 or 4. When comparing the CSF biomarker levels between these groups we found no significant differences, neither for Aβ42, t-tau or p-tau (Fig. 3b). A weak association could be found between MMSE and MTA, with lower MMSE scores among subjects with MTA grades 3 or 4 compared to those with MTA grades 1 or 2 (*p* = 0.04). However, we did not find any correlation between the results on the object recall test and the degree of MTA. Similarly, there were no correlations between either of the two cognitive read-outs and the biochemical CSF markers.

Next, we hypothesized that *APOE* could have an impact on the degree of brain atrophy. However, no association could be found between the different *APOE* genotypes and the various neuroradiological measures.

Brain tissues were available from 13 subjects. Three of these had been given a clinical diagnosis of AD, whereas the remaining ten had not been clinically diagnosed with any neurodegenerative disorder. In all 13 subjects tau pathology was seen, ranging from very sparse to Braak stage V. Pathology of Aβ was observed in twelve and of α-synuclein in two subjects, whereas pTDP43 immunoreactivity was seen in ten cases. One of the cognitively unimpaired subjects fulfilled the criteria for PART, whereas for the remaining nine cognitively unimpaired subjects AD related pathology at the low (four subjects) or intermediate (five subjects) level was observed [36]. Two of the subjects with clinical diagnosis of AD fulfilled the NIA-AA criteria for high level of AD related pathology [34, 35]. In one of those two subjects a substantial concomitant α-synuclein pathology was observed. One of the AD patients displayed an intermediate level of AD related pathology but also showed a type 1 CAA and vascular tissue alterations. None of the cases displayed any signs of hippocampal sclerosis. The outcome of the neuropathological assessment is summarized in Table 3.

**Table 3:** The outcome of the neuropathological assessment

Case #	Clinical diagnosis	Age range	BW in grams	Braak HPτ stage	Thal Aβ phase	CAA type	Braak αS stage	pTDP43	Vascular pathology	PAD	NIA-AA
1	AD	86–90	1208	3	4	1	0	1	1	AD	intermediate
2	C	96–90	1317	1	1	1	0	0	0	ADRP	low
3	C	91–96	1261	3	4	0	0	1	0	ADRP	intermediate
4	C	91–96	1415	1	0	0	3	1	0	PART	none
5	C	91–96	1410	2	1	0	0	0	0	ADRP	low
6	C	91–96	1460	b	1	2	0	0	0	ADRP	low
7	C	91–96	1347	3	5	0	0	1	1	ADRP	intermediate
8	AD	91–96	1225	5	4	2	6	1	0	AD/ DLBD	high
9	C	91–96	1370	3	4	0	0	1	1	ADRP	intermediate
10	AD	91–96	1225	5	4	2	0	1	0	AD	high
11	C	91–96	1170	4	4	0	0	1	0	ADRP	intermediate
12	C	91–96	1315	1	3	1	0	1	1	ADRP	low
13	C	9–96	1300	4	3	2	0	1	1	ADRP	intermediate

*AD* Alzheimer’s Disease, *C* cognitively unimpaired subject, *BW* brain weight, *HPt*, hyperphosphorylated tau, *Aβ* amyloid-β, *CAA* cerebral amyloid angiopathy, *aS* synuclein, *pTDP43* phosphorylated transactive DNA binding protein, *PAD* PathoAnatomical Diagnosis, *ADRP* AD related pathology, *DLBD* Diffuse Lewy body disease, *NIA-AA* National Institute on Aging–Alzheimer’s Association

## Discussion

The use of imaging and biochemical methods has become increasingly important in the diagnostic work-up for dementia investigations. Such disease biomarkers do not have a perfect sensitivity or specificity for any of the neurodegenerative disorders, but are providing supportive information. Mainly, the presence of MTA on a CT or MRI scan and decreased Aβ42 together with increased t-tau and p-tau in CSF is indicative of AD. However, as the effects of normal aging on both brain imaging [12, 13] and the CSF biomarkers [15, 16] often mimic those that are seen in the AD brain, both of these diagnostic tools are less useful in a patient population of advanced age [13, 14, 17, 18]. An overview of CSF biomarker and neuroimaging studies on such cohorts have been recently published [37].

We wanted to assess whether the same dissociation between brain pathology and cognitive function could be seen in the ULSAM cohort, consisting of men between 86 and 92 years of age. Our study setup was advantageous insofar that all participants could be recruited from this closely followed and geographically well-defined age homogenous population-based cohort [21]. As another favorable feature we could combine a broad set of different investigations - cognitive testing, brain imaging, CSF analyses and *post mortem* neuropathological evaluations - something that had not been previously performed on such an aged cohort. As for the choice of imaging method, we decided to use CT instead of MRI, since this technique is more readily available and more commonly used as a part of the routine dementia investigation of elderly patients, at least in Sweden (data from the national Swedish Dementia Registry, www.svedem.se).

For this exploratory study we aimed at including the largest possible number of still surviving subjects of the ULSAM cohort. However, similar to previous investigations, it was difficult to recruit such old individuals and the limited number of participants may have precluded us from detecting minor differences between the subgroups. Also, the included subjects were likely to be healthier than those who declined to participate - and did not represent any more advanced disease forms - why the dementia prevalence in our study was slightly lower compared to what has been reported in the general population [1, 38]. Moreover, the difference in prevalence may also be due to the fact that men display a lower dementia prevalence than women [38]. Also, other selection aspects may have had an influence on the outcome of this study. For instance, the exclusion of subjects on Coumadin could have further reduced the number of subjects with dementia as the use of this drug might be overrepresented among patients with cerebrovascular pathology.

Overall, our data are mainly confirming previous neuroradiological and CSF-biochemical observations among the elderly [39]. Moreover, the neuropathological assessments enabled us to demonstrate that the brains at these ages regularly display some degree of either Aβ or tau pathology. Most often they display a combined pathological picture that sometimes can be as severe as for the AD cases, even when no cognitive decline can be demonstrated. Also, these observations are in line with what has been previously observed [40].

As we analyzed a range of different neuroradiological and CSF parameters, we were able to assess the possible relationships between these measures. However, we could not find any correlations between any of the markers. Thus, our data suggest that there is an un-coupling between these structural and biochemical markers of brain integrity among very old individuals.

Moreover, there was only a weak correlation between cognitive performance and the degree of MTA. In addition, no correlation could be seen between cognition and the concentrations of the respective CSF biomarkers. These findings thus suggest that there also is a poor coupling between the cognitive functions and the structural / neurochemical alterations in the investigated age group.

As it has been suggested that *APOE* is associated to Aβ deposition, with *APOE ε*2 carriers displaying less pathology [41, 42], we also investigated a possible influence of *APOE* on the degree of brain atrophy. However, in this study we did not find any association between *APOE* and the various neuroradiological measures.

Taken together, our data confirm that age-related degenerative processes seem to cause tissue atrophy and aggregation of altered proteins in the brain, but without the involvement of pathophysiological processes that result in the type of cognitive decline seen in AD and other neurodegenerative disorders. Alternatively, the results may be explained by the presence of unknown protective factors in certain cognitively intact subjects, whose brains nevertheless are affected by an AD-like pathophysiology. In either way, the interpretation of an advanced MTA or of an AD-characteristic CSF profile should not necessarily be considered pathological in very old individuals.

Further research is needed to better understand which underlying factors that can explain the fundamental differences in the clinical picture between elderly individuals with and without cognitive dysfunction.

## Conclusion

Neuroradiological, biochemical and neuropathological measures of neurodegeneration do not correlate with each other in elderly men. These measures also do not reflect cognitive performance, except for the degree of MTA which in our study weakly correlated to a decreased MMSE score. Thus, biomarkers of AD are less informative in very old subjects.

## Abbreviations

AD: Alzheimer’s disease
APOE: Apolipoprotein E.
Aβ: Amyloid-beta
CAA: Cerebral amyloid angiopathy
CSF: Cerebrospinal fluid
CT: Computed tomography
ECRT: Enhanced cued recall test
GCA: Global cortical atrophy
HE: Hematoxylin eosin
HPτ: Hyperphosphorylated tau
IHC: Immunohistochemistry
LP: Lumbar puncture
MMSE: Mini mental state examination
MRI: Magnetic resonance imaging
MTA: Medial temporal atrophy
NIA-AA: National Institute of Aging and Alzheimer’s disease
PA: Posterior atrophy
PART: Primary age related tauopathy
pTDP43: Phosphorylated transactive DNA binding protein 43
ULSAM: Uppsala longitudinal study of adult men
αS: Alpha-synuclein
